# Supplementary material for: Tyrosinase Inhibitory Peptides from Enzyme Hydrolyzed Royal Jelly: Production, Separation, Identification and Docking Analysis
Source: Foods. 2023 Jun 1;12(11):2240. doi: 10.3390/foods12112240 (PMC10252203; doi:10.3390/foods12112240)
Supplement: Supplementary file 1 [file foods-12-02240-s001.zip › foods-2390042-supplementary.docx]

**Supplementary file**

**Manuscript ID: foods-2390042**

**Table S1.** Factors and levels of the Box-Behnken experiment design

| Factors | Levels | | |
| --- | --- | --- | --- |
|  | -1 | 0 | 1 |
| Solid-liquid ratio | 1：3 | 1：5 | 1：7 |
| Temperature (℃) | 30 | 50 | 70 |
| Enzyme dose (U/g) | 4000 | 7000 | 10000 |

**Inhibition rate of tyrosinase%**

**B: Temperature (℃)**

**A: Liquid-solid ratio (ml/g)**

**Inhibition rate of tyrosinase%**

**B: Temperature (℃)**

**A: Liquid-solid ratio (ml/g)**

**Inhibition rate of tyrosinase%**

**A: Liquid-solid ratio (ml/g)**

**C: Enzyme dosage (U/g)**

**Inhibition rate of tyrosinase%**

**A: Liquid-solid ratio (ml/g)**

**C: Enzyme dosage (U/g)**

**Inhibition rate of tyrosinase%**

**C: Enzyme dosage (U/g)**

**Inhibition rate of tyrosinase%**

**C: Enzyme dosage (U/g)**

**B: Temperature (℃)**

**B: Temperature (℃)**

**Figure S1.** RSM and contour maps of the effects of solid-liquid ratio (A), temperature (B) and enzyme dosage (C) on the enzymatic hydrolysis of royal jelly.
